# Advanced age and elevated cholesterol predict diabetic neuropathy in patients with type II diabetes mellitus in Southern Ethiopia

**DOI:** 10.1186/s40842-025-00241-9

**Published:** 2025-11-22

**Authors:** Fasika Merid, Habtamu Esubalew, Tamirat Gezahegn Guyo, Firdawek Getahun

**Affiliations:** 1 Department of Public Health, Arba Minch College of Health Science, Arba Minch, Ethiopia; 2 Department of Public Health, College of Medicine and Health Science, Arba Minch University, Arba Minch, Ethiopia; 3 Regional Data Management Center, South Ethiopia Public Health Institute, Jinka, Ethiopia

**Keywords:** Advanced age, Hypercholesterolemia, Predictors, Diabetic neuropathy, Type 2 diabetes mellitus, Microvascular complications

## Abstract

**Background:**

Diabetic neuropathy is the debilitating most common and expensive complication of diabetes that can cause significant mortality and morbidity, resulting from foot ulceration, lower extremity amputation, and pain that reduces the quality of life. The majority of type II diabetes mellitus patients continue to develop diabetic neuropathy despite optimal glycemic control, and the identification of predictors for diabetic neuropathy is important. Thus, this study aimed to determine the predictors of diabetic neuropathy in patients with type II diabetes mellitus in Ethiopia.

**Methods:**

A hospital-based retrospective longitudinal follow-up study was conducted from January 2012 to December 2021 in patients with type II diabetes. Simple random sampling was used to select study participants, and data were collected by reviewing the medical records. Stata version 14 was used for the statistical analysis. A bivariable and multivariable Cox regression analysis was performed to identify predictors of diabetic neuropathy. The goodness of the model fitness was checked via the Cox-Snell residual. A *p*-value less than 0.05 was used to declare the statistical significance of the variables.

**Results:**

The incidence density of diabetic neuropathy was 3.56 cases per 1,000 person months of observation, and the median time to develop diabetic neuropathy was 105 months. Advanced age (adjusted hazard ratio (AHR) 1.03 (95% CI: 1.01, 1.06)) and total cholesterol ≥ 200 mg/dl (AHR 2.52 (95% CI: 1.25, 5.09)) were statistically significant predictors of diabetic neuropathy.

**Conclusion:**

Advanced age and hypercholesterolemia were significantly associated predictors with diabetic neuropathy. Healthcare providers need to monitor cholesterol levels in order to prevent or delay diabetic neuropathy development in patients with type 2 diabetes.

## Introduction

Diabetes has reached alarming levels and remains a major public health concern [1]. Worldwide, more than half a billion people live with diabetes [1, 2]. Type 2 diabetes accounts for 96% of all diabetes cases [2]. By 2040, the prevalence of type 2 diabetes mellitus (DM) is projected to be 7,862 per 100,000 [3]. The alarming values indicate that type 2 diabetes has a worrisome future that varies according to geographical region, with above 80% of type 2 DM patients living in low and middle-income countries [4]. Complications of diabetes mellitus are distinguished as acute and chronic. For patients with diabetes, the biggest problem is the long-term complications, which include diabetic neuropathy [5].

Diabetic neuropathy is a subclinical or clinically manifested disease of the peripheral nerves that occurs as a consequence of DM without other underlying causes [6]. Diabetic neuropathy is the debilitating most common and expensive complication of diabetes that occurs in up to 50% of diabetic patients during their lifetime [7–9]. It is more common among patients with longstanding diabetes, after five years and at ten years 26% and 41% respectively have diabetic neuropathy [10]. A systematic review and meta-analysis revealed that the global prevalence of diabetic neuropathy was 35.78% among individuals with type 2 diabetes, 46% in Africa and 22% in Ethiopia among patients with diabetes [11–13]. Diabetic neuropathy can cause significant mortality and morbidity, resulting from foot ulceration, lower extremity amputation, and pain that reduces quality of life [8, 14–16]. Diabetic neuropathy is a leading cause of foot ulceration [17, 18]. During their lifetime around 25% of diabetes patients will experince foot ulceration [19]. Among diabetic neuropathy patients with foot ulceration, approximately 14 to 24% will require amputation [19]. Lower extremity amputation is 10–20% higher in patients with diabetes and more common in those with type 2 diabetes [20]. The 1 and 5 year mortality following lower limb amputation is between 10 and 50% and 30 to 80% respectively, representing a devastating impact that leads to a loss of function, financial stability, and quality of life [17]. Neuropathic pain is a major disabling consequence of diabetic neuropathy, and is estimated to affect ranging from 25 to 50% of patients with diabetes [16, 18, 21, 22]. Painful neuropathic symptoms cause significant suffering in patients, which commonly leads to anxiety, sleep disorders, depression, and a reduced quality of life and societal burden [16, 18, 23].

The incidence of diabetic neuropathy is greater in patients with T2DM [14, 15]. The most common predictors in previous studies for incidence were age, poor glycemic control, comorbid hypertension, and duration of diabetes [24–27]. The global epidemic of DM and its most common complication, diabetic neuropathy, necessitate a public health directive to address modifiable predictors with the rising urgency [15]. Early identification of diabetic neuropathy in clinical practice is essential for optimal therapeutic management and to modify any risk factors to reduce disease progression and complications [7, 14]. The majority of T2DM patients continue to develop diabetic neuropathy despite optimal glycemic control, and the identification of modifiable predictors for diabetic neuropathy is important [18]. Globally, comprehensive diabetes management has been established and practiced; all of these methods are not fully attainable, particularly in developing or low-income countries with relatively high rates of poverty [28]. Most of the studies on diabetic neuropathy originated from high-income countries [29]. Thus, this study aimed to determine the predictors of diabetic neuropathy in patients with type 2 diabetes mellitus in Ethiopia.

## Methods and materials

### Study design, period, and setting

A hospital-based retrospective longitudinal follow-up study was conducted in newly diagnosed patients with type 2 DM from 1^st^ January 2012 to 31^st^ December 2021 at Hawassa University Comprehensive Specialized Hospital (HUCSH).

### Study populations and eligibility criteria

Patients with T2DM at HUCSH were the source population. Newly diagnosed patients with T2DM who were on follow-up during the study period at HUCSH were the study population. Newly diagnosed type II diabetes mellitus patients aged 18 years and above who were on follow-up in the study setting were included in the study. Those with unknown diagnosis dates, and those who had developed diabetic neuropathy at the time of T2DM diagnosis were excluded.

### Sample size determination and sampling procedure and technique

The sample size was determined via Stata software version 14 power analysis on the basis of the following assumptions: α = 0.05, power = 80%, adjusted hazard ratio (AHR) = 2.78 from a previous study conducted [25], variability of covariates of interest = 0.5, probability of event = 0.1663, and a 0.1 proportion of withdrawals. The study’s final sample size was 201. Simple random sampling was used to select participants in a study from a sampling frame.

### Study variables

Diabetic neuropathy was the dependent variable and the independent variables were sociodemographic characteristics (age, sex, residence), clinical and treatment-related characteristics (fasting blood sugar (FBS), hypertension, aneamia, duration of diabetes, diastolic blood pressure (DBP), systolic blood pressure (SBP), proteinuria, family history of DM, high-density lipoprotein (HDL), low-density lipoprotein (LDL), triglyceride, cholesterol, cardiovascular disease (CVD), nephropathy, retinopathy, and type of anti-diabetic treatment).

### Operational definitions

Diabetic neuropathy: the presence of diabetic neuropathy diagnosed by physicians based on if there is a combination of two or more of the following sensory symptoms, decreased distal sensation, or unequivocally decreased or absent ankle reflexes and found in the patient’s medical record.

Event: diabetic neuropathy development within the follow-up period.

Censored: Those who did not develop diabetic neuropathy at the end of the study, loss to follow-up, died, or transferred out before diabetic neuropathy developing during the study period.

### Data collection procedures and quality control

The data extraction checklist included sociodemographic characteristics, clinical, and treatment-related characteristics. The tool was developed after reviewing different studies. The data were collected by reviewing the medical records of T2DM patients who were on follow-up during the study period at HUCSH, Southern Ethiopia.

Before actual data collection training was given to the data collectors on how to retrieve records and the objective of the study. A pretest was conducted, and on the basis of the findings of the pretest; an adjustment was made to the data extraction checklist. Completeness and consistency were checked for the extracted data during the data collection period, and corrections were made accordingly.

### Data processing and analysis

The data collected were entered using EpiData version 3.1 and for statistical analysis, Stata version 14 was used. Descriptive statistics, including median with interquartile range (IQR), mean with standard deviation (SD), and frequencies with percentages according to the type of variables were performed. A Kaplan-Meier (KM) survival curve was used to compare the survival time differences between categorical variables. A bivariable and multivariable Cox regression analysis was performed between the dependent and independent variables to identify predictors of diabetic neuropathy. A variable in the bivariate Cox regression analysis with a *p*-value less than 0.25 was used as a candidate for multivariable Cox regression analysis. In the multivariable Cox regression analysis, a *p*-value less than 0.05 was considered statistically significant. The mean-variance inflation factor was 1.02, which was used to check for multicollinearity. The Schoenfeld residuals test was used to check the assumption of Cox proportional hazard (Prob > chi^2^ = 0.7974). The goodness of fit of the model was checked via the Cox-Snell residual. The AHR with 95% CI and *p*-value was used to examine the strength of the association and statistical significance.

## Results

### Study participant characteristics

A total of 200 T2DM patients were included in the analysis. The study participants’ mean age was 48.73 years with an SD of ± 12.58. Almost three-fourths (72.5%) were urban residents. Among the study participants, nearly half (45%) had comorbid hypertension and 12% had cardiovascular disease. More than one-fourth (29%) of the participants had HDL cholesterol levels less than 40 mg/dl and more than one-third (35.5%) had total cholesterol levels ≥ 200 mg/dl. Among the total study participants, twenty-eight (14%) developed diabetic nephropathy. More than half (55%) of the study participants were on oral hypoglycemic agents (Table 1).

**Table 1:** Study participant characteristics

Variable	Categories	Frequency (%)
Age	Mean (SD) 48.73 ± 12.58	
Sex	Male	106 (53.00)
	Female	94 (47.00)
Residence	Urban	145 (72.50)
	Rural	55 (27.50)
Fasting blood sugar	Median (IQR) 171.5 (74.5-268.5)	
Hypertension	Yes	90 (45.00)
	No	110 (55.00)
Family history of DM	Yes	69 (34.50)
	No	131 (65.50)
Anemia	Yes	11 (5.50)
	No	189 (94.50)
Proteinuria	Positive	43 (21.50)
	Negative	157 (78.50)
Diabetic retinopathy	Yes	10 (5.00)
	No	190 (95.00)
Cardiovascular disease	Yes	24 (12.00)
	No	176 (88.00)
Triglyceride	< 150 mg/dl	122 (61.00)
	≥ 150 mg/dl	78 (39.00)
HDL	< 40 mg/dl	58 (29.00)
	≥ 40 mg/dl	142 (71.00)
LDL	< 100 mg/dl	138 (69.00)
	≥ 100 mg/dl	62 (31.00)
Total cholesterol	< 200 mg/dl	129 (64.50)
	≥ 200 mg/dl	71 (35.50)
Treatment	Oral hypoglycemic agent	110 (55.00)
	Insulin	73 (36.50)
	Both	17 (8.50)
Diabetic nephropathy	Yes	28 (14.00)
	No	172 (86.00)
Duration of T2DM	< 5 years	117 (58.50)
	≥ 5 years	83 (41.50)

### Incidence of diabetic neuropathy

**Fig. 1:**
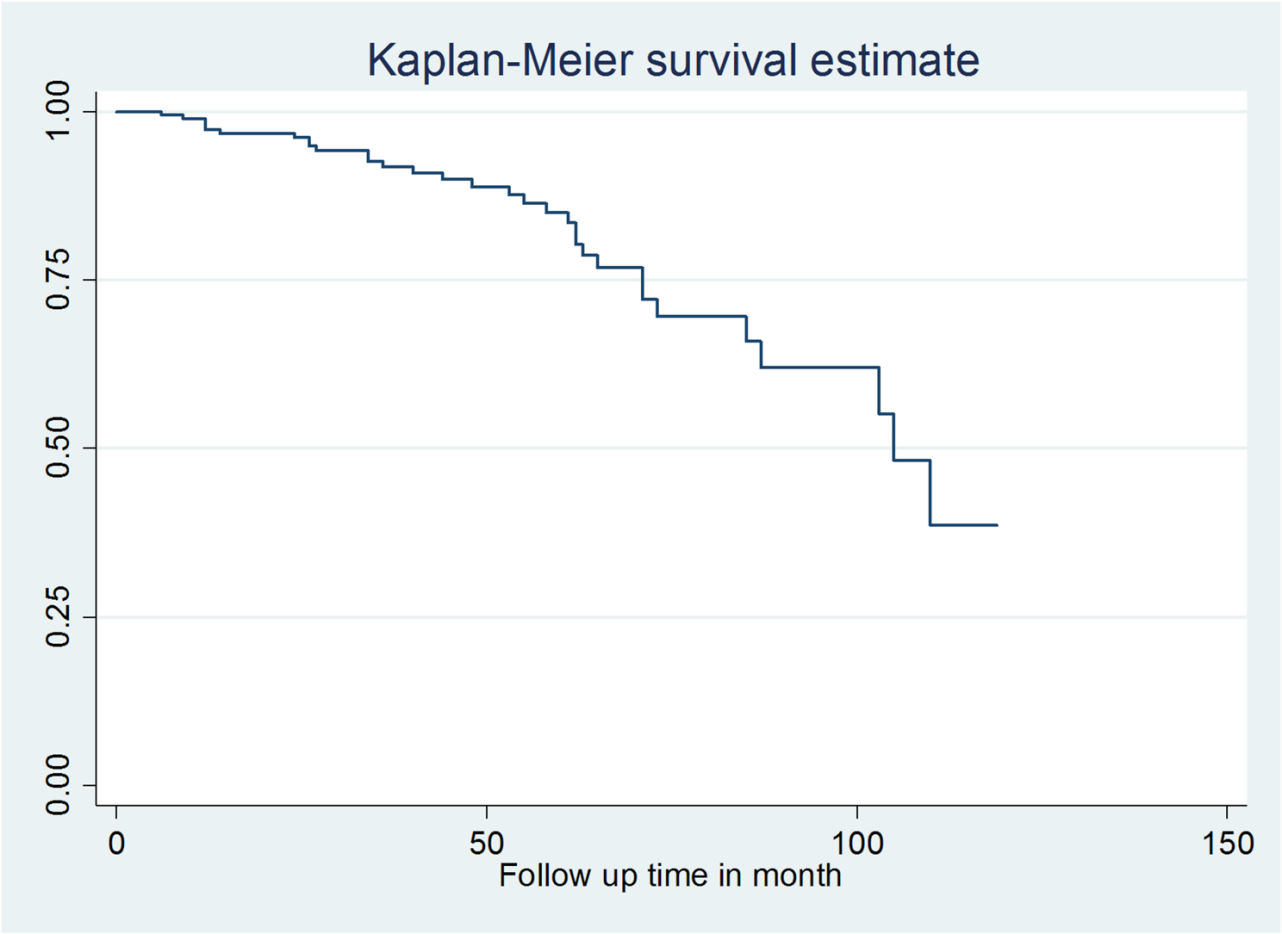
Overall Kaplan survival curve

Thirty-two (16.0%) patients with type 2 diabetes developed diabetic neuropathy with an incidence rate of 3.56 cases per 1,000 person months of observation with 95% CI (2.51, 5.03) in 8,999 person months of observation or 4.27 per 100 person year observations with 95% CI (3.01, 6.04). The median time to develop diabetic neuropathy was 105 months (Fig. 1). The cumulative survival probability was 89.56% at 48 months, 69.26% at 84 months, and 34.14% at 120 months.

### Predictors of diabetic neuropathy

Age, family history of DM, aneamia, diabetic retinopathy, cardiovascular disease, and total cholesterol were significantly associated predictors with *p*-values less than 0.25 in the bivariable Cox proportional hazard regression analysis. In the multivariable Cox proportional hazard regression analysis age and total cholesterol were statistically significant predictors with *p*-values < 0.05. For a one year increase in age the hazard of developing diabetic neuropathy increases by 3% (AHR 1.03 (95% CI: 1.01, 1.06)). Type 2 diabetes patients whose total cholesterol level was ≥ 200 mg/dl had a 2.52 fold (AHR 2.52 (95% CI: 1.25, 5.09)) higher hazard of developing diabetic neuropathy than did those whose total cholesterol level was < 200 mg/dl (Table 2).

**Table 2:** Multivariable Cox regression analysis of predictors of diabetic neuropathy

Variable	Category	Event (%)	Censored (%)	CHR (95% CI)	AHR (95% CI)
Age				1.03 (1.01, 1.06)	1.03 (1.01, 1.06)
Family history of DM	Yes	8(4.00)	61 (30.50)	0.40 (0.17, 0.94)	0.45 (0.19, 1.08)
	No	24 (12.00)	107 (53.50)	1	1
Anemia	Yes	4 (2.00)	7 (3.50)	2.07 (0.72, 6.00)	2.32 (0.79, 6.84)
	No	28 (14.00)	161 (80.50)	1	1
Diabetic retinopathy	Yes	5 (2.50)	5 (2.50)	2.49 (0.94, 6.59)	1.64 (0.52, 5.21)
	No	163 (81.50)	27 (13.50)	1	1
Cardiovascular disease	Yes	9 (4.50)	15 (7.50)	1.86 (0.85, 4.07)	1.80 (0.81, 4.01)
	No	23 (11.50)	153 (76.50)	1	1
Total cholesterol	< 200 mg/dl	14 (7.00)	115 (57.50)	1	1
	≥ 200 mg/dl	18 (9.00)	53 (26.50)	2.49 (1.24, 5.02)	2.52 (1.25, 5.09)

## Discussion

The current study aimed to determine the predictors of diabetic neuropathy in patients with type 2 diabetes mellitus. The overall incidence density of diabetic neuropathy was 3.56 cases per 1,000 person months. Age and total cholesterol were statistically significant predictors. The findings of this study have implications for physicians, program planners, policymakers, healthcare professionals, and the general public.

In our study, the incidence rate of diabetic neuropathy was 3.56 cases per 1,000 person month observation. The finding was higher than those of studies conducted in the United States of America 26.9 per 1000 person years [26], Gondar, Ethiopia 2.01 per 100 person years [25], and Northwest, Ethiopia 2.14 per 100 person year [24]. However, lower than those reported in studies done in Karachi, Pakistan 106.2 per 1000 person years [27] and Thailand 3.13 cases per 100 person months [30]. The difference might be due to the study setting, health care service provision, sample size, and duration of the follow-up period.

Advanced age strongly predicts the development of diabetic neuropathy among type 2 diabetes mellitus patients. The finding was in line with studies conducted in India [31], Indonesia [32], Gondar Ethiopia [25], China [33], and, North West Ethiopia [24]. A number of advanced age patients with diabetes mellitus progress to develop diabetic neuropathy [34]. During the aging process, several biological changes occur, which might be associated with the exacerbation with age including activation of advanced glycation end products, oxidative stress, an increase in inflammatory cells within peripheral nerve, aberrant cytokine expression, ischemia, and proinflammatory changes in the bone marrow [34, 35].

Patients with hypercholesterolemia had a higher hazard of developing diabetic neuropathy. The study finding supported by the studies done in Saudi Arabia and Bahrain [36, 37]. In patients with type 2 diabetes, there is a high incidence of dyslipidemia which is linked with diabetic neuropathy [18]. In diabetic neuropathy, lipids adversely affect the peripheral nervous system [15, 38]. The identified underlying mechanisms were the free fatty acid, plasma proteins, and oxidation of cholesterol to oxysterols that can cause injury [15, 18]. Elevated cholesterol along with glycemic control in the development of neuronal alterations should be monitored. Neurological alterations once started therapies depend on the progression of the disease [39].

### Limitations of the study

Some variables, like occupational status, alcohol consumption, cigarette smoking and body mass index were unavailable and incomplete because of the retrospective nature of the study which was based on record review and was not considered in the analysis.

## Conclusion

Age and total cholesterol were found to be independent predictors of diabetic neuropathy among patients with type 2 diabetes mellitus. Cholesterol level measurement for type 2 diabetes patients attending follow-up clinics needs to be undertaken regularly to delay and prevent the development of diabetic neuropathy, particularly in advanced-age patients.

## Data Availability

All relevant data are presented in the manuscript. However, data can be available from the corresponding author on reasonable request.
